# A review on the research progress on non-pharmacological therapy of *Helicobacter pylori*

**DOI:** 10.3389/fmicb.2023.1134254

**Published:** 2023-03-17

**Authors:** Qian Luo, Na Liu, Sugui Pu, Ze Zhuang, Hang Gong, Dekui Zhang

**Affiliations:** ^1^Department of Gastroenterology, The Second Clinical Medical College of Lanzhou University, LanZhou University Second Hospital, Lanzhou, China; ^2^Key Laboratory of Digestive Diseases, LanZhou University Second Hospital, Lanzhou, China

**Keywords:** *Helicobacter pylori*, antibiotic resistance, non-pharmacological therapy, nanomaterials, photodynamic therapy, antimicrobial peptide, phage

## Abstract

*Helicobacter pylori* is a pathogenic microorganism that mainly resides in the human stomach and is the major cause of chronic gastritis, peptic ulcer and gastric cancer. Up to now, the treatment of *Helicobacter pylori* has been predominantly based on a combination of antibiotics and proton pump inhibitors. However, the increasing antibiotic resistance greatly limits the efficacy of anti-*Helicobacter pylori* treatment. Turning to non-antibiotic or non-pharmacological treatment is expected to solve this problem and may become a new strategy for treating *Helicobacter pylori*. In this review, we outline *Helicobacter pylori*’s colonization and virulence mechanisms. Moreover, a series of non-pharmacological treatment methods for *Helicobacter pylori* and their mechanisms are carefully summarized, including probiotics, oxygen-rich environment or hyperbaric oxygen therapy, antibacterial photodynamic therapy, nanomaterials, antimicrobial peptide therapy, phage therapy and modified lysins. Finally, we provide a comprehensive overview of the challenges and perspectives in developing new medical technologies for treating *Helicobacter pylori* without drugs.

## 1. Introduction

In the 1980s, Marshall and Warren first discovered *Helicobacter pylori* (*H. pylori*) in the stomach of patients with gastritis, peptic ulceration or active chronic gastritis, which subsequently aroused significant attention in the scientific and medical community (Warren and Marshall, 1983; Marshall and Warren, 1984). *Helicobacter pylori* has been established to be a Gram-negative spiral pathogen within the gastric mucus layer or attached to the gastric epithelial cells, infecting approximately 4.4 billion people worldwide (Hooi et al., 2017). The prevalence of *H. pylori* infection is associated with socioeconomic status, level of urbanization, environmental sanitation, access to clean water and personal hygiene. Meanwhile, higher prevalence has been reported in developing countries than in developed countries (Yan et al., 2013; Hooi et al., 2017). *Helicobacter pylori* infection usually occurs during childhood and develops into chronic progressive gastritis throughout life. About 1–10% of infected individuals have clinical symptoms, including peptic ulcer disease, gastric atrophy, and intestinal metaplasia of gastric mucosa, which eventually lead to gastric cancer or mucosa-associated lymphoid tissue (MALT) lymphoma (Tshibangu-Kabamba and Yamaoka, 2021). Thus, *H. pylori* is considered an infectious disease regardless of the clinical symptoms of the infected person (Sugano et al., 2015).

*Helicobacter pylori* is classified as a gastric adenocarcinoma Group I carcinogen by the International Agency for Research on Cancer (IARC Working Group, 1994). On December 21, 2021, the US Department of Health and Human Services (HHS) released the 15th edition of the Carcinogenic Report, which listed *H. pylori* as a Class I carcinogen (U.S. Department of Health and Human Services, 2021). Therefore, the eradication of *H. pylori* is essential. The Kyoto Consensus pointed out that all people infected with *H. pylori* should receive eradication therapy unless there are other special circumstances, such as comorbidities or local reinfection (Sugano et al., 2015). According to the Maastricht VI/Florence consensus report, eradicating *H. pylori* before the stage of chronic atrophic gastritis can cure both gastritis and peptic ulcers, thereby preventing gastric cancer (Malfertheiner et al., 2022).

At present, the methods to eradicate *H. pylori* infection clinically are still limited to antibiotic-based therapies (Tshibangu-Kabamba and Yamaoka, 2021). Both the American College of Gastroenterology (ACG) Guidelines and the Toronto Consensus recommend bismuth-based quadruple therapy with a combination of proton pump inhibitor (PPI), clarithromycin, amoxicillin, and metronidazole as the first-line regimen (Fallone et al., 2016; Chey et al., 2017). The main antibiotics used clinically to eradicate *H. pylori* are amoxicillin, fluoroquinolones, rifampin, tetracycline, clarithromycin and metronidazole (Lai et al., 2022). Amoxicillin acts on the penicillin-binding proteins (PBPs) of the bacteria, which will interfere with the synthesis of the bacterial cell wall and cause the bacteria to rupture and dissolve. Fluoroquinolones can interact with bacterial DNA topoisomerases, causing irreversible damage to their chromosomes and stopping the cell division of bacteria. Rifampicin can inhibit bacterial RNA polymerase, thus blocking the process of RNA transcription and the synthesis of DNA and protein. Tetracyclines inhibit the synthesis of protein by binding to the 30S subunit of bacterial intracellular ribosomes. And clarithromycin binds to the 50S subunit of bacterial ribosomes, to hinder the synthesis of protein (Guimaraes et al., 2010; Baquero and Levin, 2021). Although the bactericidal mechanism of metronidazole has not been fully elucidated, it is generally accepted that the drug acts by the reduced nitro group (Alauzet et al., 2019).

Considering the mechanisms leading to the failure of *H. pylori* eradication, antibiotic resistance is currently the main reason. The antibiotic resistance of *H. pylori* presents three characteristics: single drug resistance, multi-drug resistance and heterogeneous drug resistance (Tshibangu-Kabamba and Yamaoka, 2021). Secondly, stomach acid can affect the effectiveness of antibiotics. For example, amoxicillin is easily degraded by stomach acid, which requires a high dose for treatment and resulting the failure of eradication due to its side effects (Zhu et al., 2022). The morphology of *H. pylori* can also affect its eradication. The coccoid forms of *H. pylori* can evade detection by the immune system and lead to the failure of antibiotic treatment (Krzyżek and Grande, 2020). Finally, the formation of biofilms can lead to the failure of *H. pylori* eradication as well (Moghadam et al., 2021).

In 2014, the antibiotic resistance had been listed as one of the three major threats to public health in the 21st century by the World Health Organization (Munita and Arias, 2016). Researches show that antibiotic resistance causes more than 7 million deaths annually worldwide, including 25,000–33,000 and 23,000 deaths in European and the United States, respectively (Baquero, 2021). According to the prediction of the World Health Organization, 10 million people may die due to the increase of antibiotic resistance by 2050 (Pulingam et al., 2022). Meanwhile, all-age mortality from antibiotic resistance is highest in some low-and middle-income countries and poses the greatest threat to human health in sub-Saharan Africa and South Asia (Murray et al., 2022). A review of antibiotic resistance in *H. pylori* shows that the global eradication rate of *H. pylori* has been declining and the increasing antibiotic resistance associated with *H. pylori* (Thung et al., 2016). The eradication rate of empirical *H. pylori* treatment has fallen below the target of 80–90%, with failure rates of 29 and 40% in the United States and Western Europe respectively, and clarithromycin resistance rates of approximately 30% in Japan and up to 50% in China (Thung et al., 2016). *H. pylori* has been listed as one of the 20 most dangerous pathogens to human health by the World Health Organization (WHO) in 2017 (Tacconelli et al., 2018). Therefore, it is imperative to develop alternative therapies to eradicate *H. pylori*, especially those resistant to multiple antibiotics. This review discussed colonization of *H. pylori* in the stomach and its virulence mechanism. Besides, the methods of non-pharmacological treatment of *H. pylori* are summarized, which provides a new treatment prospect for overcoming the problem of drug resistance of *H. pylori* in the future.

## 2. *Helicobacter pylori* colonization and its virulence mechanisms

Human gastric epithelial cells can resist the invasion of pathogens. *H. pylori* can produce various virulence factors, destroying the gastric mucosal barrier and colonizing the gastric epithelium. *H. pylori* infection can be divided into four main stages (Sharndama and Mba, 2022): 1. Adaptation to the acidic environment of the gastric cavity, 2. The movement towards the epithelial and penetration of it, 3. Adhesion and colonization of gastric epithelial cells, 4. Damage to tissue and other harmful effects.

When *H. pylori* enters the gastric cavity, it can only survive for a few minutes and must quickly migrate to the surface of the gastric epithelium (Schreiber et al., 2005). Therefore, to adapt to the acidic environment in the gastric cavity, *H. pylori* produces urease, which can hydrolyze urea into NH_3_ and CO_2_, and ammonia is used to neutralize stomach acid (Montecucco and Rappuoli, 2001). In addition, flagella can protect *H. pylori* from the stomach’s acidic environment (Saxena et al., 2020). In this way, *H. pylori* can adapt to the acidic environment of the gastric mucosa.

*H. pylori* uses flagella-mediated motility and adhesion function to penetrate the gastric mucus layer to the gastric epithelium, where it can produce adhesin and thus colonize the surface of host epithelial cells (Huang et al., 2016). The shape of spiral cells is also thought to enhance the motility of *H. pylori* through the corkscrew mechanism (Salama et al., 2013). When *H. pylori* successfully penetrates the gastric mucous layer and attaches to the gastric epithelial cells, it releases effector proteins or toxins. These virulence factors are divided into three categories (Nejati et al., 2018). The first group is present only in some *H. pylori* strains, such as the cag pathogenicity island (CagPAI) gene encoding the Cag type IV secretion system (T4SS). The second category is the virulence factors that ensure the survival of *H. pylori* under different growth conditions. Including six genes that encode the outer membrane protein(OMPS), OipA, SabA, SabB, BabA, BabC and HopZ. They are present in all strains of *H. pylori*. The last type is a genome with different gene types depending on the strain, such as VacA gene (Nejati et al., 2018). These virulence factors may disrupt the signal pathway in the host cells, thus causing chronic inflammation of the gastric mucosa (Wang et al., 2014).

Among the virulence factors of *H. pylori*, the cytotoxin-associated gene product (CagA) and vacuolar cytotoxin A (VacA) are most widely studied. CagA is a highly immunogenic protein with a molecular weight of 120–140 kDa and encoded by the CagA gene located at the end of the pathogenic island (Nejati et al., 2018). The Cag type IV secretion system (T4SS) is reportedly encoded by the cag pathogenicity island (CagPAI), and it injects CagA into host cells like a needle (Cover et al., 2020). All *H. pylori* strains possess cagA, but some are cagA positive and some cagA negative (Sharndama and Mba, 2022). CagA-positive *H. pylori* strains can stimulate the secretion of IL-8 and IL-12 in the serum of infected individuals (Eskandari-Nasab et al., 2013; Ferreira et al., 2016). Studies have shown that patients with cagA-positive *H. pylori* infection have a higher risk of developing gastric cancer or peptic ulcer disease than with cagA-negative *H. pylori* infection (Chang et al., 2018). Meanwhile, cagA-positive strains were more motile than cagA-negative strains, which indicated that cagA was also related to bacterial motility (Figura et al., 2004). Interestingly, a meta-analysis by Wang et al. found that CagA-positive strains are more pathogenic than CagA-negative strains but easier to eradicate (Wang et al., 2017).

VacA is a protein that is secreted by the type V autotransport secretion system and enters the host cell *via* endocytosis (Nejati et al., 2018).It can disrupt cell polarity, promote epithelial cell apoptosis, inhibit T-cell proliferation and affect normal function (Palframan et al., 2012). Although all *H. pylori* strains contain VacA, the vacuolating activity of the encoded cytotoxin is different (Wroblewski et al., 2010). VacA can inhibit the activation and proliferation of T-cells and B-cells and the transmission of IFN-β signal to induce macrophage apoptosis (Baj et al., 2020). At present, vacA can be divided into many genotypes, including s1, s2, m1, m2, s1m1, s1m2, s2m2 and s2m1. The VacAs1 genotype is one of the most abundant genotypes in patients with *H. pylori* infection and is associated with peptic ulcer disease (Baj et al., 2020; Keikha et al., 2020). At the same time, different vacA genotypes may be related to the severity of *H. pylori*-induced inflammation (Thi Huyen Trang et al., 2016).

In conclusion, there are many virulence factors of *H. pylori,* and their mechanisms of action are complex. Its successful colonization and pathogenicity result from the combined effect of different bacterial virulence factors.

## 3. Non-pharmacological therapy of *Helicobacter pylori*

The data and literatures are searched in the databases of Web of science, Pubmed and China national knowledge infrastructure (CNKI) using the keywords of *H. pylori*, alternative treatment and non-pharmacological treatment, respectively. Based on these collection, several methods in non-antibiotic therapies were obtained, including pharmacological therapy and non-pharmacological therapy.

Currently, the main drugs of pharmacological therapy for *H. pylori* are vaccines, traditional Chinese medicine (TCM), medicinal plants and antibiotics. The advantages and disadvantages for each type of pharmacological therapy were briefly described. Firstly, the early prophylactic vaccines can achieve a high level of protection for body as most *H. pylori* infections occur in childhood (Sousa et al., 2022). However, no successful vaccine used in clinic has yet been developed due to the powerful ability of *H. pylori* to evade the attack of host immune (Sutton and Boag, 2019). For this purpose, scientists have made efforts with only a few vaccines entering clinical trials (Zhang et al., 2022). For example, the vaccine developed by Novartis has achieved satisfactory results in phase I clinical trials, but no additional protection for patients with CagA positive strains in phase I/II study (Malfertheiner et al., 2008, 2018). Conway Biotech’s vaccine showed good activity against *H. pylori* infection in a phase III trial evaluation, but the study has been discontinued (Zeng et al., 2015). Secondly, TCM has obtained some promising results in the treatment of *H. pylori* infection (Huang et al., 2015; Kim et al., 2016; Lin et al., 2016). While the complex composition, slow antibacterial effect and ambiguous mechanism make the safety of continuous medication need to be considered in depth (Li et al., 2021). As for medicinal plants, they have potential cytotoxicity and adverse side effects, though high activity in anti-*H. pylori* (Wang, 2014). Finally, antibiotics are widely used in clinical practice because of the rapid action and broad antibacterial spectrum. On the contrary, they will lead to the disorder of intestinal microbes and an increase in drug resistance (Yang et al., 2014). Generally speaking, pharmacological therapy has unavoidable limitations in the eradication of *H. pylori* due to the problems of safety and drug resistance.

**Figure 1:**
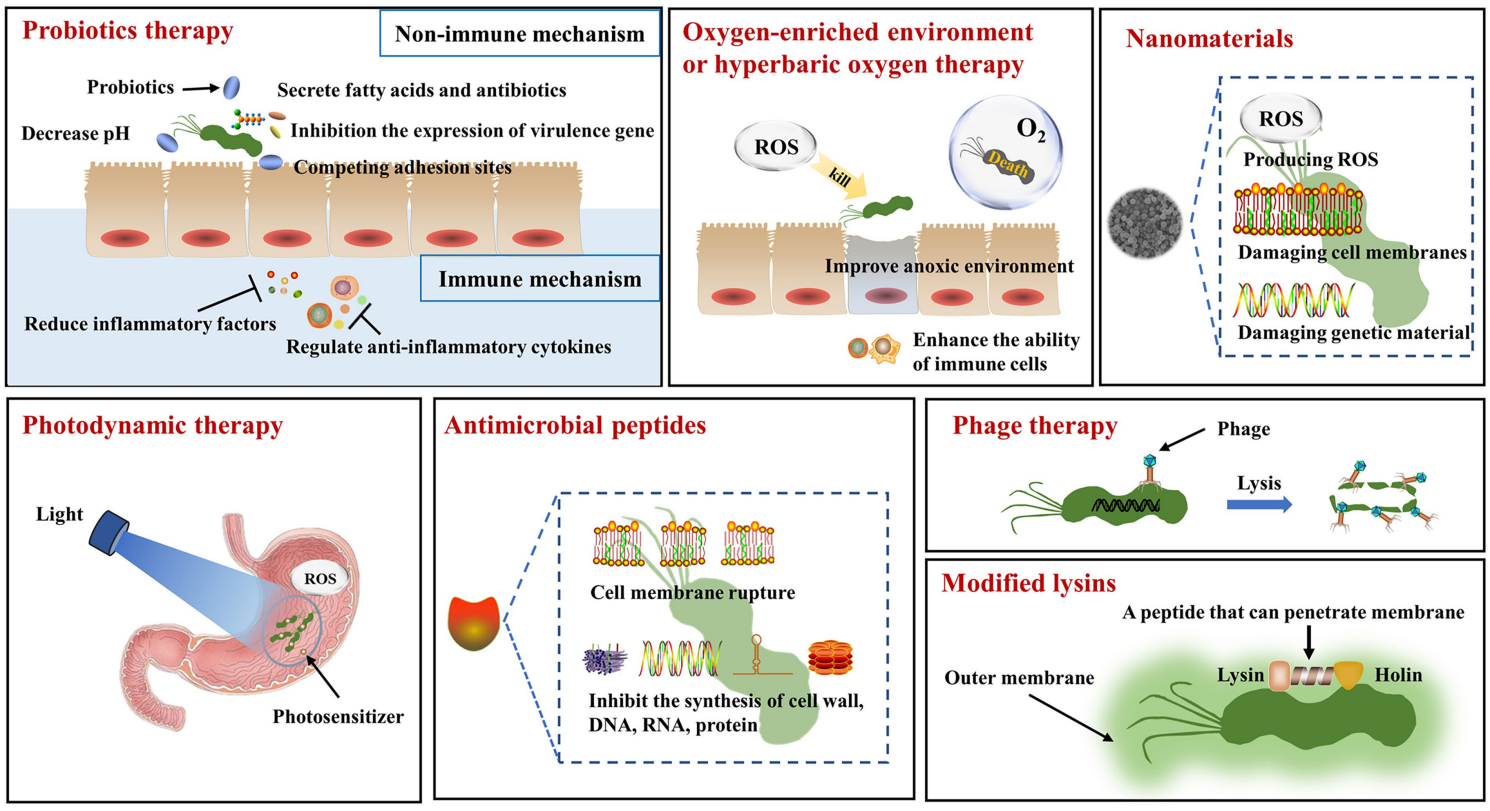
The mechanisms of non-pharmacological therapy for *Helicobacter pylori*. Probiotics therapy: In the non-immune mechanism, probiotics can secrete fatty acids and antibiotics to act on *H. pylori*, inhibit the expression of virulence gene in *H. pylori*, and compete for the adhesion sites of *H. pylori*. In the immune mechanism, probiotics can reduce the release of inflammatory factors and regulate the secretion of anti-inflammatory factors. Oxygen-enriched environment or hyperbaric oxygen treatment: Oxygen-rich environment makes it impossible for *H. pylori* to survive. Hyperbaric oxygen therapy produces reactive oxygen species that act on *H. pylori*, improving the hypoxic environment and increasing the phagocytic capacity of immune cells. Nanomaterials: Nanomaterials can generate reactive oxygen species and destroy the cell membrane and genetic material of *H. pylori*. Photodynamic therapy: The combination of light source and photosensitizer can kill *H. pylori* by producing reactive oxygen species. Antimicrobial peptides: Antimicrobial peptides can disrupt cell membranes and inhibit the synthesis of cell walls, DNA, RNA and proteins. Phage therapy: Phage specifically acts on *H. pylori* to make it lyse. Modified lysins: The genetically engineered lysins can penetrate the outer membrane of *H. pylori* and interact with it.

Thus, it is of great importance to conduct research on non-pharmacological therapies, especially in the post-antibiotic era. Although some non-pharmacological therapies need clinical testing before application, they can be effective in avoiding antibiotic resistance and its associated side effects. In the following, we carefully introduce seven main approaches for pharmacological treatments, including probiotic therapy, oxygen-enriched environment or hyperbaric oxygen therapy, antimicrobial photodynamic therapy, nanomaterials, antimicrobial peptides, phage therapy and modified lysins. Table 1 describes specific therapeutic schedules illustrated with examples. The corresponding mechanisms and the advantages and limitations for each method of non-pharmacological treatment are briefly described in Figure 1 and Table 2, respectively.

**Table 1:** Non-pharmacological therapy of *Helicobacter pylori.*

Non-pharmacological	Mechanism of action	Examples
Probiotics therapy	Immune mechanism and non-immune mechanism.	*Saccharomyces boulardii*, LactoLevure (Zhao et al., 2021; Viazis et al., 2022).
Oxygen-enriched environment or hyperbaric oxygen therapy	*H. pylori* cannot survive in an oxygen-rich environment; hyperbaric oxygen can inhibit mucosal inflammation.	Hydrogen peroxide-mediated oxygen enrichment, Hyperbaric oxygen combined with antibiotics (Gan et al., 2015; Di et al., 2020).
Antimicrobial photodynamic therapy	Produce cytotoxic reactive oxygen species to kill bacteria.	The p3SLP therapy system, LED endoscope capsule (Im et al., 2021; Luzzi and Tortora, 2022).
Nanomaterials	Generate reactive oxygen species or disrupt cell membranes, genetic material or proteins.	Ag-NPs, GNS@Ab (Amin et al., 2014; Zhi et al., 2019)
Antimicrobial peptides	Increased membrane permeability, the formation of pores, and ultimately the lysis of the microbial cell.	Cbf-K_16_, rPGLa-AM1 (Zhang et al., 2017; Jiang et al., 2020)
Phage therapy	Virulent phages or their cleaved proteins induce the lysis of host bacterial cells.	φHPE1, φHPE2, Hp φ + LF-HA (Abdel-Haliem and Askora, 2013; Cuomo et al., 2020)
Modified lysins	Lysins modified by genetic engineering can penetrate the bacterial cell membrane and target the cell wall.	The two-component lysis systems fused a hydrophobic peptide, Arlysins (Xu et al., 2020, 2021).

**Table 2:** Advantages and limitations for the non-pharmacological treatment of *Helicobacter pylori*.

Non-pharmacological	Advantages	Limitations	Reference
Probiotics therapy	Increased eradication rate, decreased side effects, less influence on gut microbiota	Low eradication rate of monotherapy	Losurdo et al. (2018), He et al. (2022), Viazis et al. (2022)
Oxygen-enriched environment or hyperbaric oxygen therapy	Enhanced ability of immune cells to phagocytize, synergistic effect with antibiotics	Different genotypes of *H. pylori* respond differently to oxygen	Al-Waili and Butler (2006), Turhan et al. (2009), Park and Lee (2013)
Antimicrobial photodynamic therapy	Targeting bacteria, killing the drug-resistant strains	Difficulty *in vitro* experiments	Luzzi and Tortora (2022), Yang et al. (2022)
Nanomaterials	High efficacy and therapeutic index, excellent synergy with antibiotics, enhanced performance of drug delivery and release	Toxicity and difficulty in degradation	Mba and Nweze (2021), Patil-Sen (2021)
Antimicrobial peptides	Significant selectivity to bacterial cells, broad-spectrum activity, cost-effective synthesis	Instability, poor bioavailability, short half-life and cytotoxicity	Li et al. (2022), Mba and Nweze (2022)
Phage therapy	Easy to isolate, high specificity, no adverse immune responses and no impact on human microbiome	Susceptible to stomach acid and digestive enzymes	Nobrega et al. (2016), Vinner et al. (2019), Anyaegbunam et al. (2022)
Modified lysins	Penetrating the outer membrane of bacteria	Producing neutralizing antibodies	Xu et al. (2020), Abdelrahman et al. (2021)

### 3.1. Probiotics therapy

Scientists from the Food and Agriculture Organization of the United Nations (FAO) and the World Health Organization (WHO) have come to a consensus that defines probiotics as “living microorganisms that, when consumed in sufficient quantities, can provide health benefits to the host”(Hill et al., 2014). Most probiotics are colonized in the human intestinal tract, and some of them, such as *Lactobacillus*, are colonized in the human stomach and directly or indirectly fight against *H. pylori* (Ji and Yang, 2020). There is already evidence that probiotics have a therapeutic effect on a variety of diseases, such as some diarrheal diseases include acute infectious diarrhea, antibiotic-associated diarrhea, *Clostridium difficile*-associated diarrhea. On top of that, there are digestive disorders, including hepatic encephalopathy, ulcerative colitis, irritable bowel syndrome, functional gastrointestinal disorders, and necrotizing enterocolitis (Yousefi et al., 2019).

Importantly, it has been shown that probiotics are also effective against *H. pylori* infection. The mechanism of probiotics in treating *H. pylori* can be divided into immune and non-immune mechanisms. The main immunological mechanism is that probiotics can reduce the release of inflammatory chemokines and regulate the secretion of anti-inflammatory cytokines by interacting with epithelial cells, thus reducing inflammation caused by *H. pylori* infection (Eslami et al., 2019; Sousa et al., 2022). In the non-immune mechanism, probiotics can compete with *H. pylori* for epithelial cell adhesion sites or inhibit the expression of adhesion-related genes in order to reduce *H. pylori* colonization (Sakarya and Gunay, 2014; Klerk et al., 2016). Probiotics can lower the pH of the environment in which *H. pylori* lives, thus reducing bacterial activity and inhibiting urease activity (Sgouras et al., 2004). Probiotics can also inhibit or kill *H. pylori* by secreting short-chain fatty acids and antibiotics (Homan and Orel, 2015). In addition, probiotics can reduce the expression of virulence genes of *H. pylori* (Urrutia-Baca et al., 2018).

In recent years, there have been many reports of integrating probiotics as adjuvant therapy into the standard treatment regimen of *H. pylori*. Firstly, probiotics can improve the eradication rate of *H. pylori*. For instance, Viazis et al. (2022) carried out a study on the combination of probiotic regimen with standard *H. pylori* eradication therapy in eight tertiary hospitals. They found the eradication rate was 92.0% in the probiotic group and 86.8% in the placebo group, with an odds ratio of 1.76 (95% CI 1.06–2.94). Secondly, probiotics can reduce the incidence of side effects. Zhao et al. conducted a clinical trial of *Saccharomyces boulardii* (*S. boulardii*) combined with quadruple therapy. It was found that the supplementation of *S. boulardii* can significantly reduce the incidence of eradication-related adverse events (AEs; OR: 0.378, 95% CI: 0.117–0.807). In particular, it reduced the duration of diarrhea (5.0 vs. 7.7 days, *p* = 0.032) and the incidence of severe diarrhea (4.7 vs. 10.1%, *p* = 0.040, 56). In addition to the above, probiotics can also reduce the effect of antibiotics on the gastrointestinal flora. He et al. studied the effect of probiotics combined with a quadruple bismuth-containing regimen on the eradication rate of *H. pylori*, gastrointestinal adverse events (GAE), and gastrointestinal microbiota. They found a lower incidence of gastrointestinal adverse events in the probiotic group than in the placebo group (23.6 vs. 37.7%), but no significant difference in eradication rates. After *H. pylori* eradication, probiotics partially counteract the effects of antibiotics on the intestinal flora and reduce fluctuations in the gastric microbiota. It can be seen that probiotics have already performed well as an adjuvant therapy (He et al., 2022).

Several systematic reviews and meta-analyses show that probiotic monotherapy has a minimal impact on the eradication rates of *H. pylori*. However, when used in supplementary therapy, probiotics are effective in increasing eradication rates with reduced side effects (Losurdo et al., 2018; Zhou et al., 2019; Zhang et al., 2020; Penumetcha et al., 2021). The Maastricht VI/Florence consensus report also states that probiotics can increase the eradication rate of *H. pylori* by reducing side effects associated with eradication therapy rather than through direct effects on *H. pylori*, but the benefit only apply to certain strains of probiotics (Malfertheiner et al., 2022). Although the Toronto guidelines do not encourage the routine use of probiotics to reduce adverse reactions or improve radical treatment rates, they are beneficial and unlikely to be harmful in certain high-risk cases (Fallone et al., 2016). Meanwhile, the American Gastroenterological Association (AGA) considers probiotics beneficial, but uncertainties about the optimal dose, timing of administration and duration of treatment should be addressed before their widespread use (Chey et al., 2017).

Although the eradication rate of probiotic monotherapy remains unsatisfactory, it is widely accepted as adjuvant therapy. Indeed, probiotics are widely thought to have huge potential; however, more data is needed to evaluate the direct efficacy of probiotics against *H. pylori*. Moreover, their effects on *H. pylori* eradication and the intestinal microflora warrant further study.

### 3.2. Oxygen-enriched environment or hyperbaric oxygen therapy

Oxygen plays a vital role in the lives of many microbes. *Helicobacter pylori* is generally considered a micro-aerobic microorganism and needs to be cultured by lowering oxygen levels. It survives in a micro-aerobic environment with an oxygen content of 5–10% (volume/volume%) in the air (Kelly, 2001; Wallace et al., 2016). Given the microaerobic environment is a prerequisite for the survival of *H. pylori*, the oxygen-enriched environment may not be conducive to the growth of *H. pylori* or even lead to its failure to survive (Di et al., 2020). For example, Jia et al. reported eradicating *H. pylori* by establishing an oxygen-enriched environment with hydrogen peroxide (Di et al., 2020), and satisfactory results were obtained *in vivo* and *in vitro* for four *H. pylori* strains, with the destruction of *H. pylori* cell membranes.

In addition, hyperbaric oxygen therapy has bactericidal or bacteriostatic effects against aerobes and anaerobes (Vatansever et al., 2013). Hyperbaric oxygen therapy can suppress pathogens or improve patient symptoms in various ways. Hyperbaric oxygen therapy can directly resist pathogenic microorganisms by forming reactive oxygen species (ROS; Memar et al., 2019). Meanwhile, hyperbaric oxygen can inhibit mucosal inflammation, improve the anoxic environment of the lesion and surrounding tissues, and enhance the ability of immune cells to phagocytize and kill bacteria (Al-Waili and Butler, 2006; Almzaiel et al., 2013; Memar et al., 2019). Hyperbaric oxygen has been reported to yield a synergistic antibacterial effect with antibiotics (Turhan et al., 2009). A Chinese study on the efficacy of hyperbaric oxygen combined with antibiotics in treating *H. pylori*-positive patients found that negative conversion increased, symptoms disappeared quickly, and the recurrence rate was low (Gan et al., 2015). These findings suggest that we can use hyperbaric oxygen or establish oxygen-enriched conditions to eradicate *H. pylori* or as an adjuvant therapy against *H. pylori*. However, some studies have found differences in the response of different genomes to oxygen pressure in *H. pylori*, suggesting that *H. pylori* may be an aerobic bacteria that requires high carbon dioxide levels to grow (Park and Lee, 2013).

In short, there are few studies on treating *H. pylori* with oxygen enrichment or hyperbaric oxygen, and the effect of oxygen concentration on *H. pylori* eradication warrants further study. At the same time, hyperbaric oxygen therapy has been popularized in most hospitals, so it is worth carrying out research on hyperbaric oxygen as adjuvant therapy combined with antibiotics to treat infection of drug-resistant strains.

### 3.3. Antimicrobial photodynamic therapy

Photodynamic therapy is a modern, non-invasive treatment method used primarily for various types of cancer treatment (Kwiatkowski et al., 2018). Given the emergence of antibiotic resistance, photodynamic therapy has become an alternative therapy for treating pathogenic microorganisms.

Antimicrobial photodynamic therapy (aPDT) uses endogenous or exogenous photosensitizers to absorb harmless visible light at appropriate wavelengths and react with oxygen molecules inside and around cells to produce cytotoxic reactive oxygen species (ROS) or singlet oxygen molecules. These cytotoxic reactive oxygen species produced *in situ* can destroy biological macromolecules, resulting in bacterial cell death (Morici et al., 2020). This method can effectively kill a variety of bacteria without causing the development of drug resistance (Al-Mutairi et al., 2018). Current evidence suggests that *H. pylori* can spontaneously produce endogenous photosensitizer porphyrin, mainly consisting of protoporphyrin IX (PPIX) and coproporphyrin I and III (CPI and CPIII), with a characteristic absorption peak at 415 nm (Battisti et al., 2017a; Battisti et al., 2017b). When the appropriate wavelength of light reacts with the photosensitive porphyrin and surrounding oxygen, it can produce cytotoxic reactive oxygen species (ROS), which can damage bacterial cells and lead to the death of *H. pylori*.

It is reported that photodynamic therapy can kill *H. pylori* while ignoring the drug resistance. Moreover, *H. pylori*-targeted photosensitizers can avoid the undesired phototoxicity to normal cells (Yang et al., 2022). Im et al. (2021) proposed an *H. pylori*-targeted photodynamic therapy system-p3SLP, which yielded significant antibacterial activity and had no adverse side effects on normal tissues and intestinal flora. Ma et al. found that a blue light-emitting diode (LED) could inhibit the proliferation of drug-resistant strains of *H. pylori in vitro* (Ma et al., 2018). Moreover, LED endoscopic capsules combined with active motion systems have been designed based on the combined action of vision and actuation to move precisely to the target area to kill *H. pylori* (Luzzi and Tortora, 2022).

Overall, much emphasis has been placed on addressing the problems caused by antibiotic resistance. Photodynamic therapy has made good progress in drug-resistant *H. pylori* strains, but additional *in vitro* and *in vivo* studies and well-designed clinical trials are needed to further prove the advantages of this treatment.

### 3.4. Nanomaterials

Nanomaterials have unique physical and chemical properties, such as small size and excellent biocompatibility, which enable them to penetrate most physiological barriers. The high surface area to volume ratio and targeted action on the lesion sites increases their interaction with pathogen biofilms while exhibiting good stability (Santos et al., 2018; Patil-Sen, 2021). Importantly, we can manipulate the size and shape of these particles and their specific ligands to improve efficacy and therapeutic index and reduce side effects (Van Giau et al., 2019; Mba and Nweze, 2021). Also, they exhibit excellent synergistic effects with antibiotics and enhancing drug delivery and release (Mba and Nweze, 2021). And nanomaterials used for the treatment of *H. pylori* can be described in two aspects: organic and inorganic nanomaterials.

The main organic nanomaterials used for the treatment of *H. pylori* are nanoemulsions, liposomes, nanostructured lipid carriers and polymeric nanoparticles. They are often used as drug delivery systems to increase the stability, release properties of drugs, as well as combat antibiotic resistance (de Souza et al., 2021). Among them, nanoemulsions are highly biocompatible and have long-term stability, which can improve the stability, solubility and bioavailability of the loaded drug (Lai et al., 2022). For example, Tran et al. have developed an delivery system of nanoemulsion encapsulated with erythromycin, which significantly enhanced the stability of erythromycin for the eradication of *H. pylori* (Tran et al., 2017). Liposomes are considered to be the most widespread nanosystems for the delivery of antimicrobial drug owing to the similar composition with cell membrane and good safety. Additionally, liposomes can integrate with other materials to exhibit good targeting specificity (Li et al., 2019; Eleraky et al., 2020). Gottesmann et al. loaded amoxicillin onto pectin-coated liposomes, which had a significant killing effect on *H. pylori*. At the same time, pectin coating enabled liposomes to target *H. pylori* (Gottesmann et al., 2020). Nanostructured lipid carriers also have biocompatibility and stability, as well as high storage capacity and encapsulation efficiency (de Souza et al., 2021). Furthermore, they are specific for *H. pylori* and do not affect the gut microbiota (Seabra et al., 2018). Sharaf et al. (2021) loaded hesperidin (Hesp) and clarithromycin (CLR) into nanostructured lipid carriers, which effectively extended the sustained release time of the two drugs, and showed effective targeting of *H. pylori* in the simulated gastric juice experiment *in vitro*. Finally, polymer nanoparticles have high loading capacity to drugs (Cardos et al., 2021), and chitosan (CS) is the most effective and versatile polymeric material from natural sources (Eleraky et al., 2020). They also exhibit excellent positioning properties in combination with other materials. For example, Arif et al. (2018) prepared Cys-CS/PMLA nanoparticles for encapsulation of amoxicillin using cysteine conjugated chitosan (Cys-CS) and polymalic acid (PMLA), which exhibited good ph-sensitive properties and delayed the release of amoxicillin in stomach acid, thereby eradicating *H. pylori* more specifically.

The inorganic nanomaterials used in the treatment of *H. pylori* are mainly metal nanomaterials, such as silver, gold, and zinc. Metal nanoparticles can kill bacteria by generating reactive oxygen species or disrupting cell membranes, genetic material or proteins (Safarov et al., 2019). They can be directly used to treat *H. pylori* (Yang et al., 2020), especially in the eradication of multidrug-resistant strains, because of their inherent antibacterial activity (Lai et al., 2022). For example, Amin et al. use *Peganum harmala* L. seed extract synthesized Ag-NPs, which showed strong anti-*H. pylori* activity *in vivo* and *in vitro*. Compared with amoxicillin and other antibiotics, drug resistance was not observed after repeated exposure for up to 10 times (Amin et al., 2014). Similarly, Zhi et al. (2019) coupled gold nanostars with cis-aconitate modified anti-*H. pylori* polyclonal antibodies to obtain the pH-sensitive gold nanostars@*H.pylori*-antibody nanoprobes (GNS@Ab), which could kill *H. pylori* by destroying the cell membrane and could enter cells to generate active oxygen and affect the metabolism of *H. pylori*. No disruption of the gut microbiota balance was observed. In addition, the combination of metal nanomaterials with materials such as hydrogels has shown excellent performance in positioning. For example, Zhang et al. encapsulated ascorbate palmitate (AP) hydrogel on the pH-responsive metal–organic framework hydrogen-generation nanoparticles (Pd(H) @ ZIF-8; Zhang et al., 2022), which could target the site of inflammation and kill the *H. pylori* effectively. Notably, it also relieves inflammation, repairs the gastric mucosa, and does not cause imbalance in the intestinal flora.

Despite nanomaterials have been widely used in medical research, the toxicity and difficulty in degradation remain problems to be solved (Patil-Sen, 2021). Besides, nanomaterials are used as delivery systems for antibiotics, which may also cause drug resistance of *H. pylori*. Therefore, it remains promising to study nanomaterials that can directly act on *H. pylori*, especially metal nanomaterials. Perhaps the next step should focus on the study of biodegradable metal nanomaterials that can maintain the balance of intestinal ecosystem. Of course, its biological safety should be evaluated before clinical transformation for *H. pylori* eradication.

### 3.5. Antimicrobial peptides

Various organisms can produce antimicrobial peptides (AMPs), which represent a component of innate immunity responsible for protecting host cells from pathogens. These peptides usually consist of < 50 amino acids and are called amphipathic peptides (Neshani et al., 2019). Due to the growing problem of antibiotic resistance, antimicrobial peptides have been used as an alternative to fight against related pathogenic microorganisms (Xu et al., 2020).

The study found that antimicrobial peptides can quickly and efficiently kill various pathogens, with a variety of mechanisms (Pérez-Peinado et al., 2018; Chen et al., 2019). Firstly, AMPs have a net positive charge, which interacts with the negative charge of the microbial cell membrane, resulting in increased membrane permeability, pore formation, and, ultimately, microbial cell lysis. In addition, AMPs can not only transfer across the cell membrane to the bacterial cytoplasm, but also further inhibit the synthesis of cell wall, DNA, RNA, protein and cell division (Brogden, 2005; Mahlapuu et al., 2016). Notably, AMPs are effective against multidrug-resistant (MDR) bacteria, and the incidence of pathogens developing resistance to AMPs is relatively low (Chung and Khanum, 2017). Also, AMPs have significant selectivity to bacterial cells, broad-spectrum activity, and low synthesis cost (Mba and Nweze, 2022).

Various AMPs, such as defensins, are present in gastric epithelial cells and they play an important role in the innate immune response to *H. pylori* infection. Although AMPs produced by gastric epithelial cells have a protective effect, *H. pylori* continues to colonize, indicating that *H. pylori* exhibits selective drug resistance to host AMPs (Nuding et al., 2013; Pero et al., 2017, 2019). In addition, there are still many disadvantages of most AMPs, such as instability, poor bioavailability, short half-life and cytotoxicity (Li et al., 2022). Natural antimicrobial peptides are unstable in the gastrointestinal tract, poor absorption and rapid metabolism, which leads to low bioavailability (Luong et al., 2020). Therefore, it is necessary to synthesize AMPs analogues or develop new technologies such as genetic engineering to effectively overcome these shortcomings (Sousa et al., 2022). Jiang et al. (2020) reported that Cbf-K_16_ had good antibacterial activity against clarithromycin-and amoxicillin-resistant *H. pylori* SS1 both *in vivo* and *in vitro*. Zhang et al. synthesized recombinant PGLa-AM1(rPGLa-AM1), which has the advantages of low toxicity and high stability with good anti-*H. pylori* activity *in vitro* and *in vivo* (Zhang et al., 2017).

Although antimicrobial peptides have certain limitations, the emergence of new technologies such as genetic engineering can effectively overcome these shortcomings. Therefore, antimicrobial peptides are widely considered to have great clinical application prospects.

### 3.6. Phage therapy

Bacteriophage (phages) are virus particles that infect bacteria. According to their relationship with host bacteria, bacteriophages can be divided into virulent (or obligately lytic) phages and temperate (or lysogenic) phages. Virulent phages recognize bacterial surfaces and inject their nucleic acids into host cells, where they then assemble, multiply, and eventually destroy bacterial cells while releasing phage progeny that infects new bacterial cells (Sulakvelidze, 2005; Muñoz et al., 2020).

Traditional phage therapy is defined as using virulent phages or their cleaved proteins to induce the lysis of host bacterial cells, thereby eliminating bacterial infection (Viertel et al., 2014). After the temperate phages bind to the host, the temperate phages are removed from the bacterial genome under environmental changes or other physiological conditions, which can eventually destroy the host cell as well (Kortright et al., 2019). Phage therapy has many advantages. For instance, phages are relatively easy to isolate and only affect the target strains since phages, and phage lyases are highly specific (Muñoz et al., 2020). In addition, phages mutate more frequently than bacteria, which can help eradicate phage-resistant bacteria (Ghannad and Mohammadi, 2012). Phages also do not require multiple doses to be effective, they rapidly replicate exponentially, and rapidly enter a death phase when the target bacteria are reduced, without causing adverse immune responses and affecting the human microbiome (Anyaegbunam et al., 2022). Phage therapy has proved effective in treating various infections and even curing chronic infections (Abedon, 2019).

To date, there are few studies on *H. pylori* specific phage. Abdel-Haliem and Askora (2013) reported that anti-*H. pylori* phages φHPE1 and φHPE2 isolated from wastewater could adapt to the acidic environment of the human stomach and exhibited high thermal stability. However, the physiological conditions inside the stomach can also hinder the ability of most phages to fight *H. pylori*. For example, the acidity of gastric juice and digestive enzymes greatly change the biological and structural composition of phages, thus reducing their proliferation and concentration at the infected site (Nobrega et al., 2016; Vinner et al., 2019). Therefore, phages have also been combined with other materials to increase stability in the stomach. For example, Cuomo et al. reported that *H. pylori*-specific lytic phage combined with lactoferrin adsorbed on hydroxyapatite nanoparticles (Hp φ + LF-HA) against *H. pylori* infection could effectively reduce bacterial colonization and related inflammatory reactions with host cells. It is worth noting that the nanoparticles (LF-HA) significantly increased the antibacterial activity of *H. pylori*-specific lytic phage (Hp φ; Cuomo et al., 2020).

Although bacteria may also resist phage and may lead to an immune system response that reduces efficacy (Principi et al., 2019), phage remains a promising treatment for *H. pylori* given the significant challenge of antibiotic resistance leading to treatment failure. The next step we should focus on the search and design of *H. pylori*-specific phage.

### 3.7. Modified lysins

The phage uses two-component lysis systems to destroy the bacterial cell wall composed of holins and endolysins (lysins), which work together to form the holin–lysin systems (Cisek et al., 2017). Holins are involved in the triggering process of host cell lysis; their function is to open holes in the host cell cytoplasmic membrane, thus providing the opportunity for endolysins to act on the cell wall (Cisek et al., 2017). Lysins are phage-encoded enzymes that lyse host bacterial cells at the end of the lytic cycle, and lysins target the peptidoglycan (PG) layer, which is an important component of the bacterial cell wall (Lai et al., 2020). Holins and lysins are widely thought to play a synergistic role and eventually destroy the host cells (Kakasis and Panitsa, 2019).

In recent years, significant inroads have been made in intracellular lysins of Gram-positive bacteria, while research on intracellular lysins of Gram-negative bacteria has barely progressed due to interference from the outer membrane (OM; Abedon, 2019). Given that *H. pylori* is a Gram-negative bacterium, it has an outer membrane outside the cell wall, which prevents lysins from destroying the cell wall when they act on the bacterium alone (Ghose and Euler, 2020). Studies have found that these lysins are easy to obtain, they can be further designed and modified by genetic engineering, and they are also easy to express and purify, and at the same time, they are suitable for industrial production (Xu et al., 2021). Therefore, in addition to discovering lysins with the inherent ability to penetrate the outer membrane, people began to design a variety of modified lysins. For example, Xu et al. (2020) reported that the two-component lysis systems of *H. pylori* phage KHP30 fused a hydrophobic peptide, which can penetrate the bacterial biofilm, thus obtaining modified lysins. The bacteriostatic experiment *in vitro* also achieved good results. Subsequently, lysins and holins combined with a polypeptide that can penetrate the outer membrane were designed, and the complex was named “arlysins”(Xu et al., 2021). *In vitro* antibacterial experiments showed that the arlysins have a strong anti-bacterial effect on *H. pylori*, leading to the perforation and destruction of its outer membrane.

The use of lysins can cause an immune response, producing cytokines and neutralizing antibodies in the body, which may cause the inactivation of lyases (Abdelrahman et al., 2021). However, developing improved lysins through new techniques, such as gene recombination, is still possible to overcome these immune responses to lysins. There are few reports on treating *H. pylori* with modified lysins, but with the continuous development of genomics, more research will be conducted in this area.

## 4. Conclusions and future perspectives

The treatment of *H. pylori* has been a great challenge. Currently, antibiotic-based therapy remains the mainstay of treatment for *H. pylori* eradication. However, due to the widespread use of antibiotics, antibiotic resistance has become a major conundrum accounting for the reduction in *H. pylori* eradication rates. There is an urgent need to develop non-antibiotic treatment strategies and prevent antibiotics abuse. Therefore, we summarized seven non-pharmacological treatments: probiotics, oxygen-rich environment or hyperbaric oxygen therapy, antibacterial photodynamic therapy, nanomaterials, antimicrobial peptide therapy, phage therapy and modified lysins. Probiotics are mainly used as an adjuvant therapy against *H. pylori*, while the eradication rate of probiotics monotherapy remains very low. Although hyperbaric oxygen therapy is widely used in treating other pathogens, there are few reports on treating *H. pylori.* For the establishment of an oxygen-enriched environment to eradicate *H. pylori*, it has been reported that different genomes of *H. pylori* have different responses to oxygen, which warrants further study. Fortunately, photodynamic therapy, antimicrobial peptides, nanomaterials, phage therapy and modified lysins can all be used to directly treat *H. pylori* infection without antibiotics, and some therapies have been shown to have a therapeutic effect on drug-resistant strains of *H. pylori*. Therefore, these methods can effectively avoid problems caused by the use of antibiotics and drug-resistant strains of *H. pylori.* However, each treatment requires preclinical and clinical studies to evaluate its efficacy and adverse reactions, and costs and patient compliance should also be considered.

## Author contributions

DZ: give the idea, revise the manuscript. QL: summarized the related literature, composed the draft of the manuscript. NL: composed the draft of the manuscript. SP, ZZ, and HG: revise the manuscript. All authors contributed to the article and approved the submitted version.

## Funding

This work was supported by the Key R&D Program of Gansu Province (grant number 20YF8FA078), Key Talent Project of Gansu Province (grant number 2022RCXM071), Upper Gastrointestinal Cancer Screening Project in Minority Areas of Gansu Province.

## Conflict of interest

The authors declare that the research was conducted in the absence of any commercial or financial relationships that could be construed as a potential conflict of interest.

## Publisher’s note

All claims expressed in this article are solely those of the authors and do not necessarily represent those of their affiliated organizations, or those of the publisher, the editors and the reviewers. Any product that may be evaluated in this article, or claim that may be made by its manufacturer, is not guaranteed or endorsed by the publisher.
